# Phosphatidic Acid Mediates the Nem1-Spo7/Pah1 Phosphatase Cascade in Yeast Lipid Synthesis

**DOI:** 10.1016/j.jlr.2022.100282

**Published:** 2022-09-20

**Authors:** Joanna M. Kwiatek, Bryan Gutierrez, Enver Cagri Izgu, Gil-Soo Han, George M. Carman

**Affiliations:** 1 Rutgers Center for Lipid Research, New Jersey Institute for Food, Nutrition, and Health, Rutgers University, New Brunswick, New Jersey, USA; 2 Department of Chemistry and Chemical Biology, Rutgers University, New Brunswick, New Jersey, USA; 3 Cancer Institute of New Jersey, Rutgers University, New Brunswick, New Jersey, USA; 4 Department of Food Science, Rutgers University, New Brunswick, New Jersey, USA

**Keywords:** phosphatidate, diacylglycerol, triacylglycerol, phosphatidate phosphatase, Pho85-Pho80, Nem1-Spo7 protein phosphatase, endoplasmic reticulum, phospholipid bilayer, reconstitution, proteoliposome, PA, phosphatidate, PAP, phosphatidate phosphatase, PC, phosphatidylcholine, PI, phosphatidylinositol, PS, phosphatidylserine

## Abstract

In the yeast *Saccharomyces cerevisiae*, the *PAH1*-encoded Mg^2+^-dependent phosphatidate (PA) phosphatase Pah1 regulates the bifurcation of PA to diacylglycerol (DAG) for triacylglycerol (TAG) synthesis and to CDP-DAG for phospholipid synthesis. Pah1 function is mainly regulated via control of its cellular location by phosphorylation and dephosphorylation. Pah1 phosphorylated by multiple protein kinases is sequestered in the cytosol apart from its substrate PA in the membrane. The phosphorylated Pah1 is then recruited and dephosphorylated by the protein phosphatase complex Nem1 (catalytic subunit)-Spo7 (regulatory subunit) in the endoplasmic reticulum. The dephosphorylated Pah1 hops onto and scoots along the membrane to recognize PA for its dephosphorylation to DAG. Here, we developed a proteoliposome model system that mimics the Nem1-Spo7/Pah1 phosphatase cascade to provide a tool for studying Pah1 regulation. Purified Nem1-Spo7 was reconstituted into phospholipid vesicles prepared in accordance with the phospholipid composition of the nuclear/endoplasmic reticulum membrane. The Nem1-Spo7 phosphatase reconstituted in the proteoliposomes, which were measured 60 nm in an average diameter, was catalytically active on Pah1 phosphorylated by Pho85-Pho80, and its active site was located at the external side of the phospholipid bilayer. Moreover, we determined that PA stimulated the Nem1-Spo7 activity, and the regulatory effect was governed by the nature of the phosphate headgroup but not by the fatty acyl moiety of PA. The reconstitution system for the Nem1-Spo7/Pah1 phosphatase cascade, which starts with the phosphorylation of Pah1 by Pho85-Pho80 and ends with the production of DAG, is a significant advance to understand a regulatory cascade in yeast lipid synthesis.

## Graphical abstract

**Figure:**
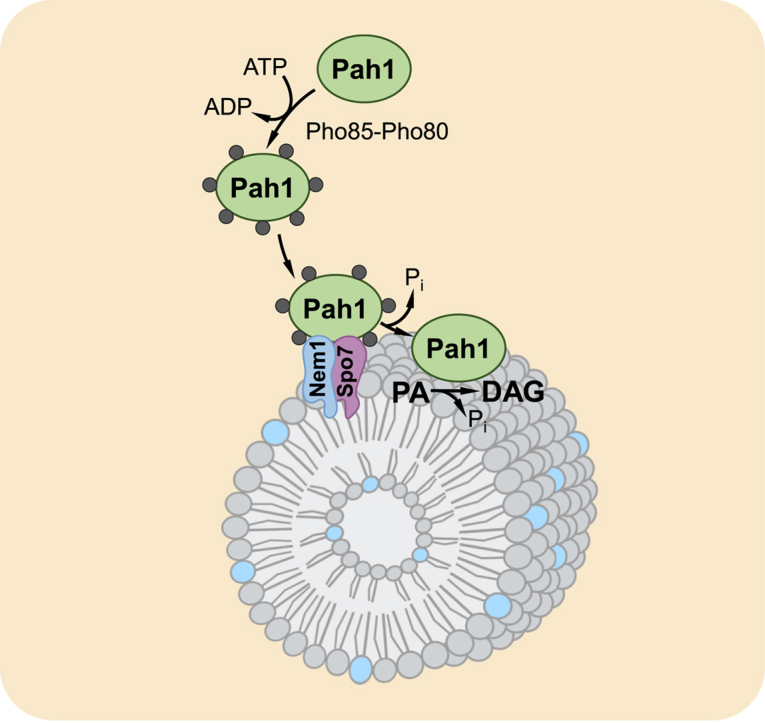

In the budding yeast *Saccharomyces cerevisiae*, the major phospholipids of the nuclear/ER membrane include phosphatidylcholine (PC), phosphatidylethanolamine (PE), phosphatidylinositol (PI), and phosphatidylserine (PS) (1, 2). In the primary de novo pathway (Fig. 1), these phospholipids are derived from the branch point intermediate phosphatidate (PA), which itself is synthesized in the nuclear/ER membrane (6, 7, 8, 9, 10, 11, 12). The PA is activated with CTP to form another branch point intermediate, the liponucleotide CDP-diacylglycerol (DAG) (13, 14). In the nuclear/ER membrane, CDP-DAG donates its phosphatidyl moiety to inositol to form PI (15, 16, 17) or to serine to form PS (18, 19, 20, 21). The PS synthesized in the nuclear/ER is transported to the mitochondrial membrane (22, 23) and decarboxylated to form PE (24, 25), which is then transported to the nuclear/ER membrane and subjected to three successive steps of methylation to form PC (26, 27, 28, 29). The synthesis of these phospholipids primarily occurs when cells are actively growing (e.g., exponential phase) and need to make cellular membranes, but as cells progress into stasis (e.g., stationary phase), the intermediate PA is primarily channeled into the storage lipid triacylglycerol (TAG) via DAG (30, 31). The TAG produced in this pathway is then stored in cytoplasmic lipid droplets (32, 33). The DAG produced from PA (34, 35) may also be used by ethanolamine and/or choline auxotrophic mutants (27, 28, 29, 36, 37, 38, 39, 40) defective in CDP-DAG-dependent synthesis of PS, PE, and/or PC by way of the CDP-ethanolamine (41, 42, 43, 44) and/or CDP-choline (45, 46, 47, 48) branches of the Kennedy pathway. Of note, phospholipids and TAG, as well as lipid droplets, may be produced at the inner nuclear membrane in genetically modified cells (49).

**Fig. 1:**
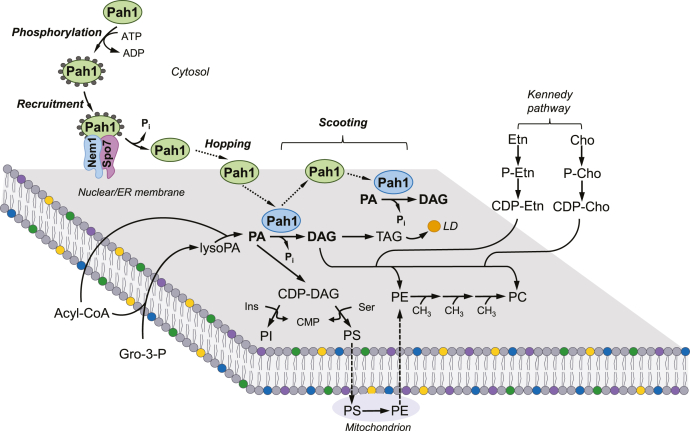
Model for the phosphorylation/dephosphorylation-mediated regulation of Pah1 PAP location and mode of action of the enzyme at the nuclear/ER membrane via the hopping and scooting modes. Pah1 in the cytosol is phosphorylated by several protein kinases. The phosphorylated enzyme (*gray circles*) translocates to the nuclear/ER membrane via its recruitment and dephosphorylation by the Nem1-Spo7 protein phosphatase complex. Dephosphorylated Pah1 hops onto the membrane, scoots along the surface to bind its substrate, and catalyzes the dephosphorylated PA to produce DAG, which can be utilized in the formation of TAG stored in lipid droplets (LDs). Following the PAP reaction, Pah1 scoots along the membrane until it recognizes a PA molecule for another round of reaction. Pah1 catalyzing the PAP reaction is designated with blue color. PA is also converted to CDP-DAG for the synthesis of the membrane phospholipids PI, PS, phosphatidylethanolamine (PE), and PC. The phospholipids PE and PC may also be derived from DAG via the CDP-ethanolamine and CDP-choline branches of the Kennedy pathway when cells are supplemented with ethanolamine and/or choline. Further details of the lipid synthesis pathways may be found elsewhere (3, 4, 5). PA, phosphatidate; PAP, phosphatidate phosphatase; PC, phosphatidylcholine; PI, phosphatidylinositol; PS, phosphatidylserine.

The bifurcation of PA between CDP-DAG and DAG is a crucial point in lipid synthesis regulation, and among the enzymes that utilize PA at this branch point, the *PAH1*-encoded Mg^2+^-dependent PA phosphatase (PAP) (also known as Pah1) (35) has emerged as a key regulator of PA consumption (2, 50, 51, 52, 53, 54). In fact, disturbances in the bifurcation of PA as mediated by Pah1 give rise to a variety of lipid-based abnormalities (e.g., nuclear/ER membrane expansion, lipodystrophy, and fatty acid-induced lipotoxicity) and defects in cellular physiology that lead to apoptosis and reduction in chronological life span (2, 3, 4, 55). Thus, knowledge of Pah1 regulation is of fundamental importance. It is known that Pah1 is regulated by genetic and biochemical mechanisms (2, 51, 52, 53, 54, 56). Pah1 expression is regulated by growth phase and nutrient status; increased expression as mediated by nutrient depletion in stationary phase is coincident with PA utilization for TAG synthesis, whereas reduced expression as mediated by nutrient sufficiency in exponential phase is coincident with PA utilization for phospholipid synthesis (31, 57). The PAP activity of Pah1 is modulated by lipids (58, 59), nucleotides (60), and the phosphorylation status (61, 62, 63, 64, 65, 66, 67, 68, 69, 70). For example, PAP activity is stimulated by CDP-DAG and PI (58), whereas the enzyme activity is inhibited by sphingoid bases (59) and the nucleotides ATP and CTP (60). Multiple protein kinases phosphorylate Pah1 (63, 64, 65, 66, 67, 68, 69), whereas the phosphorylated enzyme is dephosphorylated by the Nem1 (catalytic subunit)-Spo7 (regulatory subunit) phosphatase complex (61, 70, 71). Whereas some of its phosphorylations (e.g., by Pho85-Pho80 and Rim11) inhibit PAP activity (64, 69), the dephosphorylation by Nem1-Spo7 stimulates activity (64, 70). Pah1 phosphorylation and dephosphorylation, respectively, are generally associated with reduced and elevated enzyme function (54).

The most important factor responsible for Pah1 function is its cellular location, which is controlled by its phosphorylation and dephosphorylation (54) (Fig. 1). After its expression, Pah1 in the cytosol is phosphorylated by multiple protein kinases (54). The phosphorylation stabilizes the enzyme (72) but makes it nonfunctional because it is sequestered in the cytosol apart from its substrate PA that resides in the nuclear/ER membrane. Phosphorylated Pah1 translocates to the nuclear/ER membrane through its recruitment and dephosphorylation by the Nem1-Spo7 protein phosphatase complex (61, 62, 63, 64, 65, 71, 73, 74, 75, 76). Dephosphorylated Pah1 hops onto the membrane via its N-terminal amphipathic helix (73), scoots along the surface to recognize its substrate PA, and catalyzes the dephosphorylated PA to produce DAG (77). Following the PAP reaction, Pah1 scoots along the membrane for another round of reaction (77).

The aim of this work is to develop a proteoliposome model system that mimics the Nem1-Spo7/Pah1 phosphatase cascade that occurs at the nuclear/ER membrane. In a previous study, we showed that a liposome composed of PC, PE, PI, PS, and PA, which mimics the phospholipid composition of the nuclear/ER membrane (1, 78, 79), provides a suitable model for the action of unphosphorylated Pah1 to hop and scoot along the membrane surface and catalyze the PAP reaction (77). Herein, we significantly advanced this in vitro model for studying the dephosphorylation of Pah1 at the membrane surface by the Nem1-Spo7 complex reconstituted in the PC/PE/PI/PS/PA liposomes. We discovered that the PAP substrate PA stimulates the Nem1-Spo7 complex-mediated dephosphorylation of Pah1 and demonstrated that the Nem1-Spo7/Pah1 phosphatase cascade, starting with the phosphorylation of Pah1 and ending with the generation of DAG from PA, was reconstituted in the Nem1-Spo7 proteoliposome system.

## Materials and Methods

### Materials

Avanti Polar Lipids and Analtech were the sources of lipids and silica gel GHL TLC plates, respectively. Bio-Rad was the source of molecular mass protein standards and reagents for protein assay, SDS-PAGE, and Western blotting. InstantBlue protein stain was from Expedeon. GE Healthcare was the source of the enhanced chemifluorescence Western blotting detection kit, Sephadex G-50 superfine, IgG-Sepharose, Q-Sepharose, and polyvinylidene difluoride paper. MilliporeSigma was the source of ammonium molybdate, bovine serum albumin, Triton X-100, and rabbit anti-protein A antibody (product P3775, lot 025K4777). PerkinElmer Life Sciences and National Diagnostics, respectively, were the sources of radiochemicals and scintillation-counting supplies. Thermo Fisher Scientific was the source of malachite green, alkaline phosphatase-conjugated goat anti-rabbit IgG antibody (product no. 31340, lot number: NJ178812), and Pierce strong anion exchange spin columns and protein concentrators 30K. Qiagen was the source of nickel-nitrilotriacetic acid-agarose resin. Anti-Spo7 antibody was previously produced in New Zealand White rabbits (80). Wako Chemicals supplied Phos-tag™ Acrylamide AAL-107. All other chemicals were reagent grade.

### Purification of enzymes

The strains and plasmids used for the purification of enzymes are listed in Table 1. Protein A-tagged Nem1-Spo7 protein phosphatase complex was purified from *S. cerevisiae* strain RS453-expressing plasmids YCplac111-*GAL1/10*-*NEM1*-PtA and pRS313-*GAL1/10*-*SPO7* by affinity chromatography with IgG-Sepharose (85) with minor modifications (70). His_6_-tagged Pah1 and Pah1-D398E expressed from plasmids pGH313 and pGH313-D398E, respectively, in *Escherichia coli* strain BL21(DE3)pLysS were purified by affinity chromatography with nickel-nitrilotriacetic acid-agarose (35), followed by ion exchange chromatography with Q-Sepharose (70). Tandem affinity purification-tagged Pah1 expressed from plasmid pGH452 in *S. cerevisiae* strain SS1132 was purified by affinity chromatography with IgG-Sepharose, followed by anion exchange chromatography and size-exclusion chromatography (86). His_6_-tagged Pho85-Pho80 protein kinase complex was purified from *E. coli* strain BL21(DE3)-expressing plasmids EB1164 and EB1076 by affinity chromatography with nickel-nitrilotriacetic acid-agarose (84). The purified enzyme preparations were analyzed by SDS-PAGE and judged to be highly purified; the enzymes were stored at −80°C.

**Table 1:** Strains and plasmids used in this study

Strain or Plasmid	Genotype or Relevant Characteristics	Source or Reference
Strain		
***Saccharomycescerevisiae***		
RS453	*MAT***a** *ade2-1 his3-11,15 leu2-3,112 trp1-1 ura3-52*	(81)
SS1026	*pah1*Δ*::TRP1* derivative of RS453	(61)
***Escherichia coli***		
BL21(DE3)pLysS	F^-^*ompT hsdS*_*B*_ (*r*_*B*_^-^*m*_*B*_^-^) *gal dcm* (DE3) pLysS	Novagen
BL21(DE3)	F^-^*ompT hsdS*_*B*_ (*r*_*B*_^-^*m*_*B*_^-^) *gal dcm* (DE3)	Invitrogen
Plasmid		
YCplac111-*GAL1/10*-*NEM1*-PtA	*NEM1*-PtA under control of *GAL1/10* promoter inserted in *CEN/LEU2* plasmid	(61)
pRS313-*GAL1/10*-*SPO7*	*SPO7* under control of *GAL1/10* promoter inserted in *CEN/HIS3* plasmid	(82)
pGH313	*PAH1* coding sequence inserted into pET-15b	(35)
pGH313-D398E	pGH313 containing the D398E mutation in the *PAH1* coding sequence	(83)
EB1164	*PHO85-*His_6_ derivative of pQE-60	(84)
EB1076	*PHO80* derivative of Psbeta	(84)

### Preparation of Nem1-Spo7 proteoliposomes

Nem1-Spo7 proteoliposomes were prepared by the size-exclusion chromatography as described by Mimms *et al*. (87) with minor modifications (88, 89, 90). Unless otherwise indicated, the dioleoyl derivatives of PC, PE, PS, PA, and soybean PI were used in this work. Under most conditions used in this study, the phospholipid composition of liposomes was PC/PE/PI/PS/PA (33.75:22.5:22.5:11.25:10 mol %). Additional liposomes were made of PC/PE/PI/PS (37.5:25:25:12.5 mol %), PC/PE/PI/PS/ThioPA (33.75:22.5:22.5:11.25:10 mol %), and PC/PE/PI/PS/DAG (33.75:22.5:22.5:11.25:10 mol %). The mol % of PA in the liposome composed of PC/PE/PI/PS/PA was calculated using the following formula, mol %_PA_ = (PA [molar])/(PA [molar] + PC [molar] + PE [molar] + PI [molar] + PS [molar]) × 100. Briefly, chloroform was evaporated from phospholipid mixtures under a stream of nitrogen to form a thin film, and residual solvent was removed in vacuo. Phospholipids were resuspended in 280 mM octyl glucoside to a final phospholipid concentration of 20 mM. The octyl glucoside/phospholipid-mixed micelles were then mixed with 9 μg of Nem1-Spo7 complex solubilized in 1.7 mM Triton X-100. The final molar ratios of octyl glucoside to Triton X-100 and octyl glucoside to phospholipids were 164:1 and 14:1, respectively. The reconstitution mixture (137 μl) was applied to and eluted from a Sephadex G-50 superfine column (1 × 4 cm) equilibrated at 4°C with a chromatography buffer consisting of 25 mM Tris-HCl (pH 8.0), 250 mM NaCl, and 10 mM 2-mercaptoethanol at 4°C. The presence of phospholipids and the Nem1-Spo7 complex, respectively, in the column fractions was analyzed by primuline staining of lipid spots on a TLC plate and Western blotting with anti-protein A and anti-Spo7 antibodies. Relative amounts were determined by fluorimaging; the image intensities were quantified with ImageJ software. The size of the proteoliposomes in the peak fraction, which we used in these studies, was determined by light scattering using a Brookhaven Instruments Particle Size Analyzer. Proteoliposomes were stored for no longer than one week at 4°C.

### Lipid analysis of Nem1-Spo7 proteoliposomes

Lipids were extracted from proteoliposomes by the method of Bligh and Dyer (91). Phospholipids and DAG, respectively, were resolved by one-dimensional TLC on silica gel plates using the solvent systems chloroform/ethanol/water/triethylamine (30:35:7:35, v/v) (92) and hexane/diethyl ether/glacial acetic acid (40:10:1, v/v) (93). The resolved lipids were stained with 0.5% primuline. The amounts of PA and DAG in the proteoliposomes were determined from standard curves of each lipid on TLC plates. Fluorimaging using a Storm 860 Molecular Imager (GE Healthcare) was used to acquire fluorescence signals from the plates; the intensities of the images were analyzed by ImageJ software. The identity of the lipids was confirmed by comparison with the migration of authentic standards.

### Nem1-Spo7 protein phosphatase assay

Nem1-Spo7 phosphatase activity was measured at 30°C by following an increase in electrophoretic mobility of Pah1 in SDS-PAGE (70, 94) or by following the release of ^32^P_i_ from [^32^P]Pah1 (200-1,400 cpm/nmol) (70). The reaction mixture contained 100 mM sodium acetate (pH 5.0), 10 mM MgCl_2_, 1 mM DTT, 0.2 μM phosphorylated Pah1, and Nem1-Spo7 proteoliposomes in a total volume of 20 μl. In the nonradioactive assay, the reaction was terminated with Laemmli sample buffer (95) followed by SDS-PAGE in the absence and presence of the Phos-tag reagent (70, 94). In the radioactive assay, the reaction was terminated by the addition of 20% trichloroacetic acid and 0.4 mg/ml bovine serum albumin. The mixture was cooled on ice for 15 min and then centrifuged for 20 min at 15,000 *g* to precipitate the radioactive substrate. The supernatant containing ^32^P_i_ was measured for radioactivity by scintillation counting. One unit of Nem1-Spo7 phosphatase activity was defined as the amount of enzyme that catalyzed the formation of 1 nmol phosphate per min. The Nem1-Spo7 phosphatase reactions, which were conducted in triplicate at 30°C, were linear with time and protein concentration. To prepare [^32^P]Pah1, *E. coli*-expressed unphosphorylated Pah1 was phosphorylated by the Pho85-Pho80 protein kinase complex using 100 μM [γ-^32^P]ATP (5,000–10,000 cpm/pmol) as described previously (64). [^32^P]Pah1 was purified by Q-Sepharose chromatography to remove the Pho85-Pho80 protein kinase (70).

### PAP assay

PAP activity was measured at 30°C for 15 min by following the release of water-soluble P_i_ from chloroform-soluble PA; the P_i_ produced in the reaction was measured with malachite green-molybdate reagent (96, 97). The reaction mixture contained 50 mM Tris-HCl (pH 7.5), 1 mM MgCl_2_, enzyme protein, and the PA-containing liposomes (77) in a final volume of 10 μl. Enzyme assays were conducted in triplicate, and the average SD of the assays was ± 5%. All enzyme reactions were linear with time and protein concentration. One unit of enzymatic activity was defined as the amount of enzyme that catalyzed the formation of 1 nmol of product per minute.

### SDS-PAGE and Western blotting

SDS-PAGE (95) and Western blotting (98, 99) with polyvinylidene difluoride membrane were performed by standard procedures. Phos-tag™ AAL-107 (20 μM) and MnCl_2_ (100 μM) were added to 5% polyacrylamide gels for analysis of the phosphorylation state of Pah1. The samples for Western blotting were normalized to total protein loading. The membrane was cut and the upper and lower portions, respectively, were probed with rabbit anti-protein A (2 μg/ml) and rabbit anti-Spo7 (1 μg/ml) antibodies. The goat anti-rabbit IgG antibody conjugated with alkaline phosphatase was used at a dilution of 1:4,000. Immune complexes were assayed with the enhanced chemifluorescence Western blotting substrate. Fluorescence signals from the blots were visualized by fluorimaging with a Storm 865 Molecular Imager (GE Healthcare) and image intensities were analyzed with ImageJ software.

### Preparation of ThioPA

ThioPA (C18:1) was synthesized as described by Bonnel *et al.* (100) with some modifications. Details on the synthesis and characterization of this phospholipid are found in the Supporting information.

### Data analysis

Microsoft Excel software was used for the statistical analysis of the data; the *P* values < 0.05 were taken as a significant difference. SigmaPlot software was used to analyze kinetic data.

## Results

### Reconstitution of the Nem1-Spo7 protein phosphatase complex into phospholipid vesicles

**Fig. 2:**
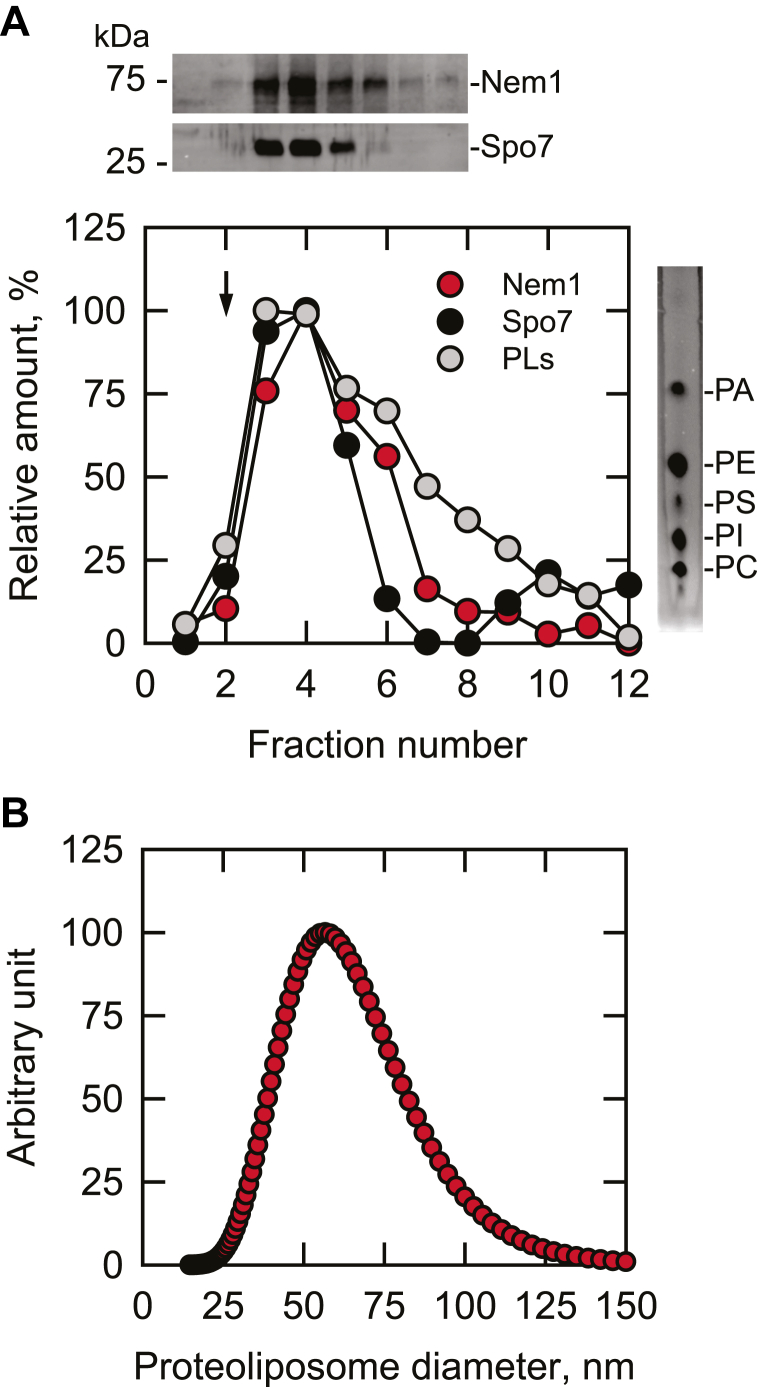
Preparation and analysis of Nem1-Spo7 proteoliposomes. A: octyl glucoside/phospholipid micelles were mixed with Triton X-100/Nem1-Spo7 complex micelles, followed by size-exclusion chromatography with Sephadex G-50 superfine. The elution fractions (0.5 ml) were analyzed for Nem1 and Spo7 by Western blotting (*upper*) with anti-protein A and anti-Spo7 antibodies, respectively, and for total phospholipids (PLs) by TLC and primuline staining (*right*). The amounts of Nem1, Spo7, and phospholipids were quantified by ImageJ software; the amount of each component in the peak fraction was set at 100%. The phospholipid composition of the peak fraction was analyzed by TLC; the positions of the individual phospholipids are indicated (*right*). The *arrow* in the figure indicates the position of the void volume. B: the peak fraction of Nem1-Spo7 proteoliposomes was subjected to particle size measurement by light scattering.

We sought to reconstitute the Nem1-Spo7 phosphatase complex into PC/PE/PI/PS/PA vesicles to mimic the in vivo environment of the complex in the nuclear/ER membrane (1, 78, 79) to recruit and dephosphorylate Pah1 (61, 62, 70, 71, 73). It has been shown that Pah1 PAP activity on PA in this phospholipid composition is higher than the enzyme activity on simple PC/PA phospholipid vesicles or Triton X-100/PA-mixed micelles (77). For the preparation of the Nem1-Spo7 proteoliposomes, we utilized the chromatographic method of Mimms *et al.* (87) that had been applied to the reconstitution of the membrane-associated phospholipid biosynthetic enzymes glycerol-3-phosphate acyltransferase (88), PI synthase (89), and PS synthase (90) into unilamellar phospholipid vesicles. Triton X-100/Nem1-Spo7 complex micelles (70, 71) were mixed with octyl glucoside/phospholipid micelles and fractionated by size-exclusion chromatography with Sephadex G-50 superfine. As expected (87, 88, 89, 90), the Nem1-Spo7 proteoliposomes were eluted from the column near the void volume, and the peak fractions of phospholipids, Nem1, and Spo7 were coincident (Fig. 2A). The TLC analysis of the proteoliposomes confirmed that the vesicles were composed of PC, PE, PI, PS, and PA (Fig. 2A, *right*), and the relative amounts of the phospholipids were estimated from their input amounts. The peak fractions of Triton X-100 and octyl glucoside micelles, which are well separated from the proteoliposomes, were contained in the later elution fractions that are not shown in Fig. 2A (87, 88, 89, 90). Light scattering analysis of the Nem1-Spo7 proteoliposomes in the peak fractions, which were used in these studies, indicated the average diameter of 60 nm (Fig. 2B), a value within the range of vesicle sizes (40–90 nm) of reconstituted glycerol-3-phosphate acyltransferase, PI synthase, and PS synthase (88, 89, 90). Disruption of the proteoliposomes with Triton X-100 did not result in a significant increase in Nem1-Spo7 phosphatase activity, indicating that the phosphatase complex is asymmetrically reconstituted with its active site located outside of the vesicle. Proteoliposomes stored at 4°C maintained Nem1-Spo7 phosphatase activity for at least one week.

### Nem1-Spo7 proteoliposomes catalyze the dephosphorylation of Pah1

The fidelity of the Nem1-Spo7 proteoliposomes to catalyze the dephosphorylation of Pah1 was examined by two different assays. In the first assay, the protein phosphatase activity was measured by following a change in the electrophoretic mobility of Pah1 in SDS-PAGE (70, 94, 101). In the second assay, the enzyme activity was measured by following the release of ^32^P_i_ from ^32^P-labeled Pah1 (70). As a substrate of Nem1-Spo7, two forms of phosphorylated Pah1 were used in this study: 1) Pah1 prepared from yeast (62, 102, 103, 104, 105, 106, 107, 108, 109, 110, 111, 112, 113, 114), which is endogenously phosphorylated by multiple protein kinases and 2) (63, 64, 65, 66, 67, 68) Pah1 heterologously expressed in *E. coli* and phosphorylated in vitro by the Pho85-Pho80 protein kinase (64). Pho85-Pho80, which phosphorylates seven sites on Pah1, has strong regulatory effects on the protein location, stability, and PAP activity (54, 62, 63, 64, 70, 72).

**Fig. 3:**
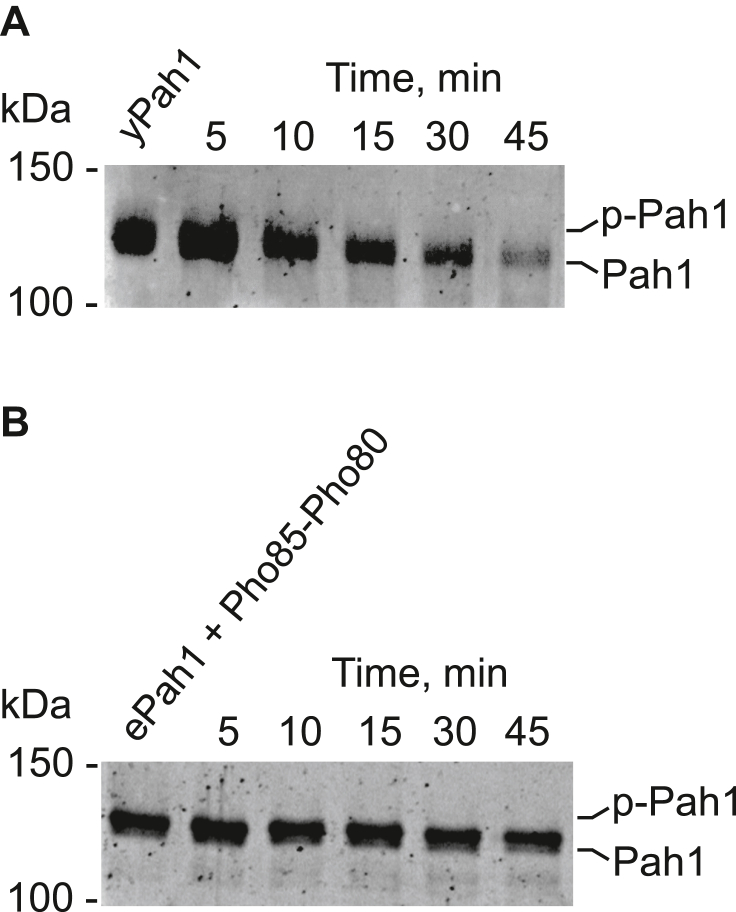
Nem1-Spo7 proteoliposomes catalyze the time-dependent dephosphorylation of Pah1. Nem1-Spo7 proteoliposomes were incubated for the indicated time periods with purified Pah1 endogenously phosphorylated in yeast (*yPah1*) (A) or *Escherichia coli*-expressed and purified Pah1 phosphorylated by Pho85-Pho80 (*ePah1 + Pho85-Pho80*) (B). Following the incubations, the samples were subjected to SDS-PAGE using a 5% polyacrylamide gel followed by staining with Coomassie blue. The positions of phosphorylated (*p-Pah1*) and dephosphorylated Pah1 and molecular mass standards are indicated. The data shown is representative of three separate experiments.

**Fig. 4:**
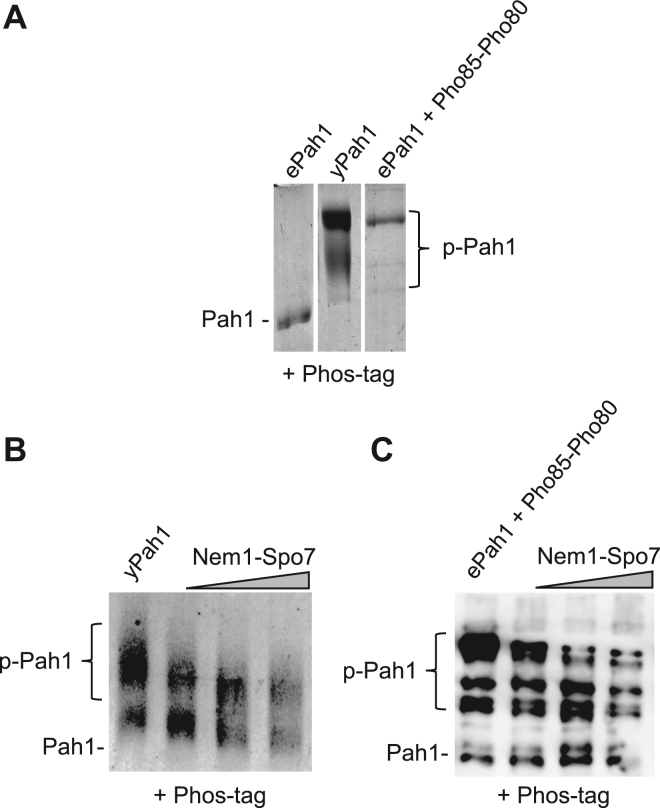
Pah1 dephosphorylation is dependent on the amount of Nem1-Spo7 proteoliposomes. Purified Pah1 endogenously phosphorylated in yeast (*yPah1*) (A, B), Pah1 expressed and purified from *Escherichia coli* (*ePah1*) (A), and *E. coli*-expressed and purified Pah1 phosphorylated by Pho85-Pho80 (*ePah1 + Pho85-Pho80*) (A, C) were subjected to SDS-PAGE using a 6% polyacrylamide gel containing 20 μM Phos-tag and 100 μM MnCl_2_. The phosphorylated forms of Pah1 (B, C) were incubated for 45 min with increasing amounts of Nem1-Spo7 proteoliposomes. The resolved proteins were stained with Coomassie blue. The positions of phosphorylated (*p-Pah1*) and dephosphorylated forms of Pah1 are indicated. The data shown is representative of three separate experiments.

In the electrophoretic mobility shift assay, the reconstituted Nem1-Spo7 catalyzed the time-dependent dephosphorylation of Pah1 phosphorylated in vivo (Fig. 3A) and in vitro (Fig. 3B) as indicated by an increase in the mobility of the protein. To better distinguish the phosphorylated and dephosphorylated forms of Pah1, we examined its mobility in the polyacrylamide gel containing the Phos-tag reagent, which traps phosphorylated proteins (115) and retards their electrophoretic mobility (70, 101) (Fig. 4A). Using the Phos-tag polyacrylamide gel, we could more readily discern changes in electrophoretic mobility and showed that the dephosphorylation of endogenously phosphorylated Pah1 (Fig. 4B) and Pah1 phosphorylated by Pho85-Pho80 in vitro (Fig. 4C) is dependent on the amount of the Nem1-Spo7 proteoliposomes. In addition, the dephosphorylation of Pah1 resulted in its degradation as reflected by the reduction of protein abundance (Fig. 4B, C).

**Fig. 5:**
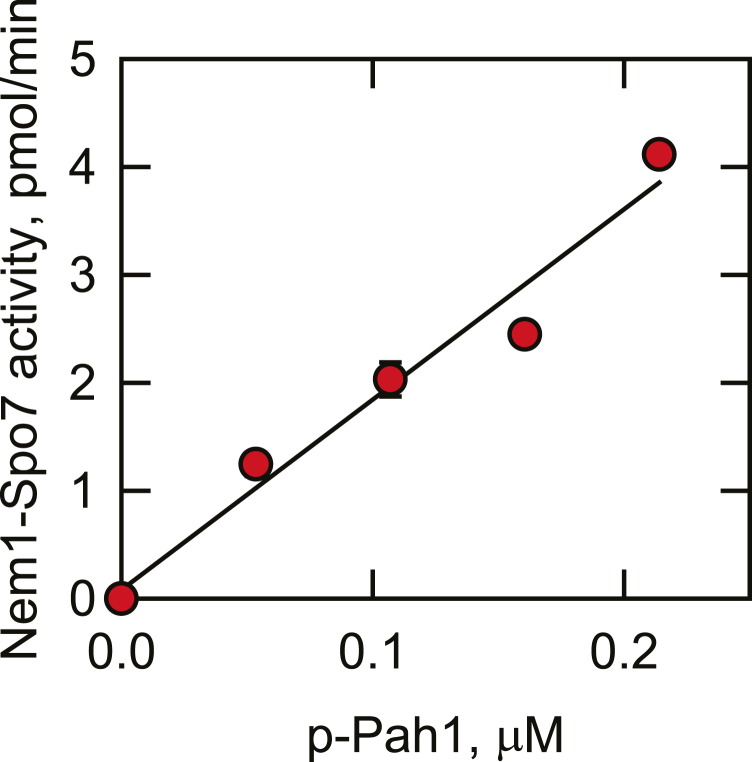
The protein phosphatase activity of Nem1-Spo7 proteoliposomes is dependent on the concentration of phosphorylated Pah1. *Escherichia coli*-expressed and purified unphosphorylated Pah1 was phosphorylated by Pho85-Pho80 with [γ-^32^P]ATP. The Nem1-Spo7 protein phosphatase activity was measured for 10 min by following the release of ^32^P_i_ from the indicated concentrations of [^32^P]Pah1. The data shown in the figure is the average of three independent experiments ± S.D. (*error bars*). The error bars are hidden behind some of the symbols.

In the radioactive assay for Nem1-Spo7 activity, which is more sensitive and quantitative, a ^32^P-labeled substrate was prepared by phosphorylating the *E. coli*-expressed unphosphorylated Pah1 by Pho85-Pho80 with [γ-^32^P]ATP. The amount of ^32^P_i_ released from [^32^P]Pah1 by Nem1-Spo7 was measured by scintillation counting after the removal of both phosphorylated and dephosphorylated forms of the protein by trichloroacetic acid precipitation (70). The Nem1-Spo7 phosphatase activity was linear over a 10-min incubation period and dependent on the concentration of phosphorylated Pah1 (Fig. 5). The radioactive assay was not performed on endogenously phosphorylated Pah1 owing to the cumbersome nature of purifying radiolabeled Pah1 from yeast cells. Overall, Nem1-Spo7 phosphatase activity in the proteoliposome system was dependent on time, the amount of the phosphatase complex, and the concentration of phosphorylated Pah1. The dephosphorylation of Pah1 was not observed when the phosphatase reaction was performed with liposomes without the reconstituted Nem1-Spo7 complex.

### PA stimulates Nem1-Spo7 phosphatase activity

**Fig. 6:**
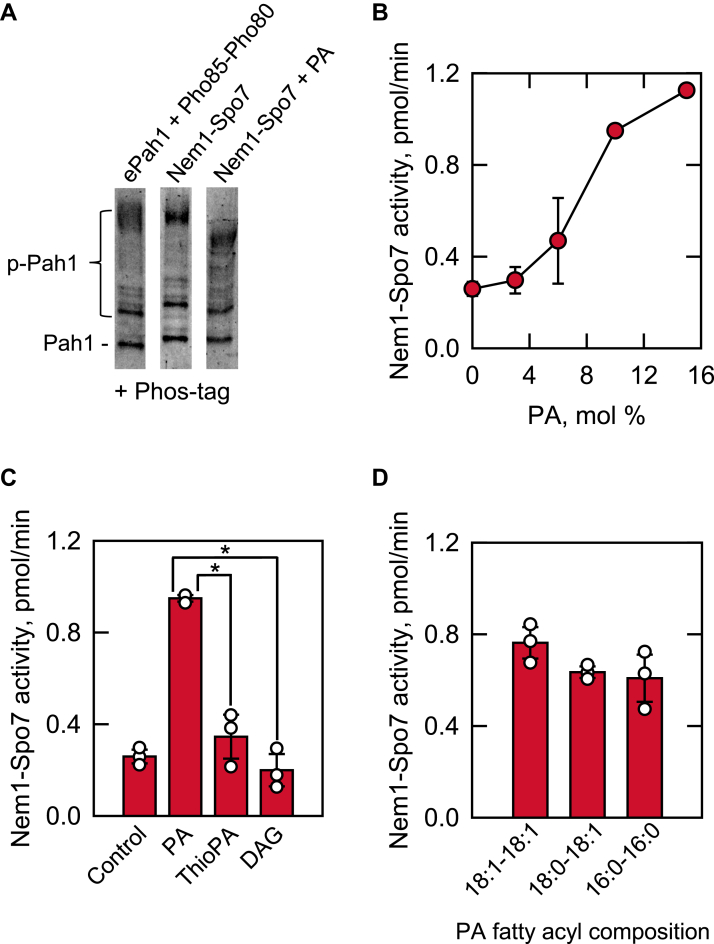
Nem1-Spo7 protein phosphatase activity is stimulated by PA in proteoliposomes. The Nem1-Spo7 complex was reconstituted into liposomes with or without 10 mol % PA, ThioPA, or DAG, or with 10 mol % PA with different fatty acyl compositions. The *Escherichia coli*-expressed and purified unphosphorylated Pah1 was phosphorylated by Pho85-Pho80 (*ePah1 + Pho85-Pho80*) with unlabeled ATP (A) or [γ-^32^P]ATP (B–D). The Nem1-Spo7 protein phosphatase activity was measured for 45 min by following the increase in the electrophoretic mobility of Pah1 visualized by staining with Coomassie blue of a 6% SDS-polyacrylamide gel containing 20 μM Phos-tag and 100 μM MnCl_2_ (A) or the release of ^32^P_i_ from [^32^P]Pah1 (B–D). The data shown in (A) is representative of three experiments, whereas the data in (B–D) are the average of three experiments ± S.D. (*error bars*). The individual data points are shown in (C, D). ∗, *P* < 0.05 versus PA-containing Nem1-Spo7 proteoliposomes. *Control*, no PA in proteoliposomes. PA, phosphatidate.

The Nem1-Spo7 proteoliposomes contained PA because it is a component of the nuclear/ER membrane (1, 78, 79) and the substrate for the PAP reaction (34). The PA concentration in the nuclear/ER membrane is largely controlled by Pah1 PAP activity (33, 35), and thus, we considered whether the PA concentration affects the Nem1-Spo7 activity on Pah1. To address this question, Pah1 phosphorylated by Pho85-Pho80 was incubated with Nem1-Spo7 proteoliposomes prepared with and without 10 mol % PA, and its dephosphorylation was assessed by electrophoretic mobility in Phos-tag SDS-PAGE. Compared with Nem1-Spo7 phosphatase activity in the presence of PA, the enzyme activity was lower in the absence of PA as reflected by a less increase in Pah1 mobility (Fig. 6A). The stimulatory effect of PA on Nem1-Spo7 phosphatase was further examined using the radioactive assay with Pah1 phosphorylated by Pho85-Pho80 with [γ-^32^P]ATP. The phosphatase complex was reconstituted in a series of phospholipid vesicles containing varying amounts of PA. Compared with Nem1-Spo7 activity in the absence of PA, the enzyme activity was stimulated by the phospholipid in a cooperative dose-dependent manner (Fig. 6B). Analysis of the data yielded a Hill number of ∼1.5. The Nem1-Spo7 phosphatase activity in the presence of 10 mol % PA was 3.6-fold greater than the enzyme activity in the absence of PA (Fig. 6B, C). In contrast to PA, thioPA (1, 2-diacyl-sn-glycero-3-phosphorothioate) containing a sulfur atom in place of an oxygen in the phosphate headgroup did not show a significant stimulatory effect on Nem1-Spo7 phosphatase activity (Fig. 6C). Similarly, the PAP product DAG substituted for PA in the proteoliposome did not stimulate the Nem1-Spo7 phosphatase activity (Fig. 6C, *control*). The stimulatory effect of PA was not affected by the composition of its fatty acyl groups, which include two saturated (16:0 to 16:0), two monounsaturated (18:1 to 18:1), or one saturated and one unsaturated (18:0-18:1) fatty acyl chains (Fig. 6D). Overall, these results indicate that the stimulatory effect of PA on Nem1-Spo7 phosphatase is governed by the phosphate headgroup but not by the fatty acyl groups of the phospholipid.

### The Nem1-Spo7/Pah1 phosphatase cascade is reconstituted with proteoliposomes

**Fig. 7:**
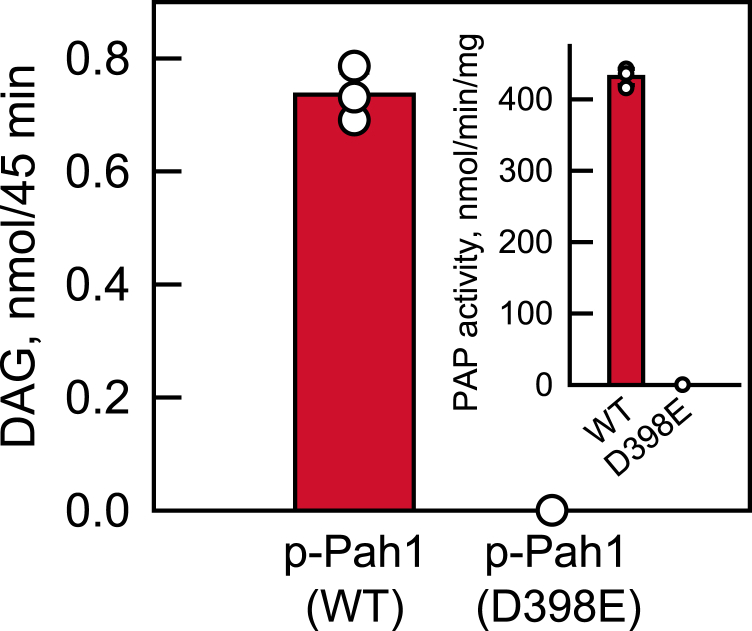
Reconstitution of the Nem1-Spo7/Pah1 phosphatase cascade in proteoliposomes. The Nem1-Spo7 complex was reconstituted into liposomes with 10 mol % PA. *Escherichia coli*-expressed and purified unphosphorylated WT or D398E forms of Pah1 were phosphorylated by Pho85-Pho80 with unlabeled ATP. The phosphorylated forms of Pah1 were incubated with the Nem1-Spo7 proteoliposomes for 45 min. Following the incubation, the proteoliposomes were collected, the lipids extracted, and the amounts of PA and DAG were analyzed by TLC. *Inset*, unphosphorylated WT and D398E forms of Pah1 were assayed for PAP activity by following the release of P_i_ from PA using liposomes composed of PC/PE/PI/PS/PA. The surface concentration of PA within the liposomes was 10 mol %. The data shown in the figure is the average of three independent experiments ± S.D. (*error bars*). The error bars are hidden behind the symbols. The individual data points are shown. PA, phosphatidate; PAP, phosphatidate phosphatase.

After confirming that Nem1-Spo7 proteoliposomes are active on phosphorylated Pah1, we sought to establish the phosphatase cascade in the reconstituted assay system. For this experiment, the Nem1-Spo7 complex was reconstituted in phospholipid vesicles containing 10 mol % PA. The proteoliposomes were incubated with Pah1 phosphorylated by Pho85-Pho80 with unlabeled ATP. The reaction mixture was incubated for 45 min at pH 5.0 to allow for the maximum Nem1-Spo7 activity on phosphorylated Pah1 and to compensate for the reduced PAP activity of dephosphorylated Pah1 at pH 5.0 when compared with its optimum at pH 7.0–7.5 (35, 116). An intermediate pH between the optimums for Nem1-Spo7 and Pah1 was not used for the assay because the former activity drops precipitously at pH above 5.0 (70). The amounts of PA and DAG in the proteasomes were determined at the 0- and 45-min time intervals to assess the course of the PAP reaction. After the 45-min incubation, the amount of DAG produced was 0.7 nmol (Fig. 7), corresponding to 17% of PA dephosphorylation from its total vesicle concentration of 4.2 nmol. Considering the accessible amount of PA in the vesicles, its conversion to DAG would be 34% based on the equal distribution of the phospholipid between the inner and outer leaflets. As expected, DAG was not produced from the reaction when PA was omitted from the Nem1-Spo7 proteoliposomes. Moreover, the catalytic site mutant Pah1-D398E (83) (Fig. 7, *inset*) did not produce DAG in the reconstituted assay system. Taken together, these results demonstrate the control of PAP activity in the reconstituted Nem1-Spo7/Pah1 phosphatase cascade.

## Discussion

In the yeast *S. cerevisiae*, Pah1 is a key regulator in lipid synthesis; it controls the bifurcation of PA between DAG and CDP-DAG for the synthesis of the neutral storage lipid TAG and membrane phospholipids, respectively (2, 50, 51, 52, 53, 54) (Fig. 1). The physiological function of Pah1 is largely controlled by its cellular location, which is mediated by phosphorylation and dephosphorylation (54). Phosphorylated Pah1 is sequestered in the cytosol apart from its nuclear/ER membrane-associated substrate PA, whereas dephosphorylated Pah1 associates with the membrane to catalyze the PAP reaction to generate DAG (54). Thus, the master regulators of Pah1 function are the protein kinases that phosphorylate Pah1 in the cytosol and the nuclear/ER membrane-associated Nem1-Spo7 protein phosphatase complex that recruits and dephosphorylates the enzyme at the membrane (61, 62, 63, 64, 65, 70, 71, 73, 74, 75, 76). In this work, we developed a model system to examine Pah1 phosphorylation by Pho85-Pho80, the protein kinase that has the greatest effect on Pah1 function (54, 64), and its dephosphorylation by the membrane-associated Nem1-Spo7 protein phosphatase complex.

We successfully reconstituted functional Nem1-Spo7 complex into phospholipid vesicles composed of PC/PE/PI/PS/PA, which constitutes the nuclear/ER membrane bilayer (1, 78, 79). The phospholipid vesicles afford a surface for PAP activity that rivals that of Triton X-100/PA-mixed micelles (77). The average size (60 nm) of the Nem1-Spo7 proteoliposomes prepared by size-exclusion chromatography was similar to that of other proteoliposomes reconstituted with lipid metabolic enzymes prepared by the same method (88, 89, 90). Moreover, the lack of latent Nem1-Spo7 activity upon proteoliposome disruption with detergent indicated that the active site of Nem1 is located at the external side of the vesicle. This model system permitted analysis of the Nem1-Spo7/Pah1 phosphatase cascade starting with the phosphorylation of Pah1 by Pho85-Pho80 and ending with the production of DAG.

Analyzing Nem1-Spo7 proteoliposomes with and without PA revealed that the protein phosphatase activity is stimulated by the PAP substrate. The stimulatory effect of PA was governed by the nature of the phosphate headgroup. For example, thioPA, the phosphorothioate analog of PA, did not stimulate Nem1-Spo7 activity. Parenthetically, thioPA is not a substrate for the PAP reaction (100). Additionally, DAG lacking the phosphate moiety of a phospholipid molecule did not stimulate the protein phosphatase activity. The fatty acyl moiety of PA did not have a significant effect on its stimulatory effect on Nem1-Spo7 activity. Likewise, the PA fatty acyl groups do not affect Pah1 PAP activity (77). The mechanism by which PA stimulates Nem1-Spo7 phosphatase activity is unclear at present. We consider that the stimulatory effect on the enzyme activity might be governed by direct interaction (117) of PA with Nem1 and/or Spo7 and/or by the effect of PA on the membrane bilayer environment (118, 119, 120, 121). Additional work is required to address these notions.

The stimulatory effect of PA on Nem1-Spo7 activity was an unexpected finding that has implications for the regulation of the phosphatase cascade affecting the PA/DAG balance and lipid synthesis. We envisage a scenario where the cell senses elevated PA levels that fuel the recruitment and dephosphorylation of Pah1 for its function on the membrane. The activation of Pah1 in turn reduces PA levels through TAG synthesis controlled by its PAP activity. Reduced PA levels would signal the opposite effects such as reduced Nem1-Spo7 activity, Pah1 hyperphosphorylation, and reduced PAP activity. This situation, however, may be an oversimplification of the complex regulation that occurs in vivo with multiple lipid biosynthetic enzymes (e.g., glycerol-3-phosphate and lysoPA acyltransferases, DAG kinase, and phospholipase D) that affect PA levels (2, 3, 4, 122).

In addition to being a phospholipid intermediate in lipid biosynthetic pathways (Fig. 1), PA itself functions as a signaling molecule in various cellular functions. In mammalian cells, PA activates cell growth and proliferation, vesicular trafficking, secretion, endocytosis, and even hair growth (123, 124, 125, 126, 127, 128, 129, 130). In plants, PA is implicated in seed germination and stress responses to low temperature, salinity, and drought (125, 126). In bacteria, PA plays a role in signaling and biofilm formation (131). In yeast, PA is required for suppression of the growth and membrane trafficking defects in the Sec14 PI/PC binding protein mutant (132, 133, 134) and in Spo20-mediated fusion of vesicles with the prespore membrane during sporogenesis (135, 136). Most germane to the control of lipid synthesis in yeast is the role of PA in the expression of lipid synthesis genes via the Henry (Opi1/Ino2-Ino4) regulatory circuit (2, 3, 4, 137). PA, along with Scs2, has the ability to sequester the Opi1 repressor at the nuclear/ER membrane, permitting the Ino2-Ino4 complex-mediated transcriptional activation of UAS_INO_-containing phospholipid synthesis genes (2, 3, 4, 137). Reduced PA levels permit Opi1 to dissociate from the nuclear/ER membrane and translocate into the nucleus where it interacts with Ino2 to attenuate transcription of UAS_INO_-containing genes (2, 3, 4, 137).

The Pah1-mediated control of PA plays an important role in the transcriptional regulation of lipid synthesis genes (e.g., *INO1*, *INO2*, *CHO1*, and *OPI3*) via the Henry regulatory circuit (61, 62, 138, 139). We posit that the PA-mediated regulation of the Nem1-Spo7 phosphatase complex must be involved in this regulation. Interestingly, the fatty acyl species of PA do not stimulate the Nem1-Spo7 and PAP activities but affect the interaction of Opi1 with the phospholipid; the PA species 34:1, the most abundant PA species in *S. cerevisiae* (33), mediates expression of the UAS_INO_-containing *INO1* gene (139). In addition, the Opi1 repressor function is also affected by the fatty acyl chain length of PA; Opi1 binding to PA is favored with its C16- over C18-chain length (140).

The reconstitution of the Nem1-Spo7/Pah1 axis is a significant advance in modeling a regulatory cascade in lipid synthesis. This proteoliposome system will permit well-defined studies to examine the structure-function relationships of Nem1, Spo7, and Pah1 as well as the phosphorylation-mediated interactions of Pah1 with the Nem1-Spo7 complex at the membrane surface. In fact, in a recent study, the system provided important information on Nem1-Spo7 activity on Pah1 phosphorylated by Pho85-Pho80 and glycogen synthase kinase homolog Rim11, which showed a preference for dephosphorylating target sites of the Pho85-Pho80 protein kinase (69).

## Data Availability

All data are contained within the article or supporting information.

## Supplemental Data

This article contains supplemental data (100).

## Conflict of Interest

The authors declare that they have no conflicts of interest with the contents of this article.
