# Supplementary material for: Phosphatidic Acid Mediates the Nem1-Spo7/Pah1 Phosphatase Cascade in Yeast Lipid Synthesis
Source: J Lipid Res. 2022 Sep 20;63(11):100282. doi: 10.1016/j.jlr.2022.100282 (PMC9587005; doi:10.1016/j.jlr.2022.100282)

Phosphatidic acid mediates the Nem1-Spo7/Pah1 phosphatase cascade in yeast lipid synthesis

Joanna M. Kwiatek[a], Bryan Gutierrez[a,c], Enver Cagri Izgu[a,c,d], Gil-Soo Han[a,b], and George M. Carman[a,b,1]

[a]Rutgers Center for Lipid Research, New Jersey Institute for Food, Nutrition, and Health, [b]Department of Food Science, [c]Department of Chemistry and Chemical Biology, and [d]Cancer Institute of New Jersey, Rutgers University, New Brunswick, New Jersey 08901

Synthesis and characterization of ThioPA (DiC 18:1)

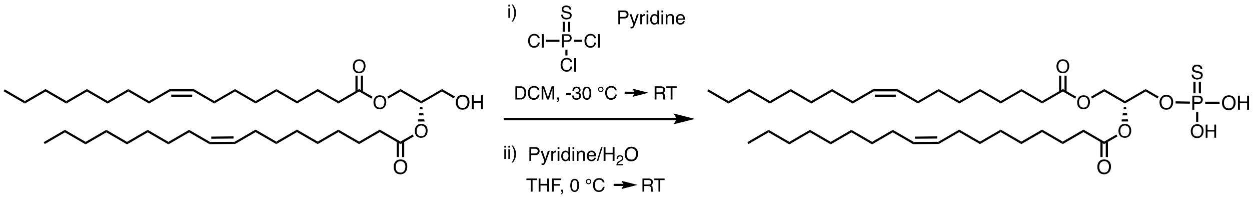

Reagents

1-2-dioleoyl-*sn*-glycerol was purchased from Avanti Lipids. Thiophosphoryl chloride, pyridine, tetrahydrofuran, 7 M ammonium hydroxide, and molybdenum blue spray were purchased from Millipore-Sigma.

Solvents

Dichloromethane (DCM) and chloroform were purchased from Millipore Sigma. Methanol (MeOH) was purchased from Fisher Scientific. Deuterated solvents were purchased from either Cambridge Isotope Laboratories or Millipore Sigma. Deuterated solvents contained 0.05% (v/v) TMS as a secondary internal reference. Water was deionized and filtered to a resistivity of 18.2 ΩM with a Milli-Q® Plus water purification system (Millipore, Massachusetts).

Methods

All glassware was oven-dried before use. Purification of ThioPA (DiC 18:1) was performed using a Büchi Reveleris® flash chromatography system equipped with a FlashPure EcoFlex Diol (50 µm spherical) column and an evaporative light scattering detector. Nuclear Magnetic Resonance (NMR) spectroscopic analyses was carried out using a Bruker Avance Neo 500 MHz spectrometer. ^{1}H, ^{13}C, and ^{31}P NMR spectra were acquired at 500, 126, and 201 MHz. Chemical shifts (δ) for the ^{1}H NMR spectrum was referenced to $(C\boldsymbol{H}_3)_4Si$ at δ = 0.00 ppm or to $CHCl_3$ at δ =

7.26 ppm. The ^{13}C NMR spectrum was referenced to ***C***DCl_3 at δ = 76.27 ppm. The ^{31}P NMR spectrum was referenced externally (using a co-axial NMR tube) to 1-palmitoyl-2-oleoyl-sn-glycero-3-phosphocholine (POPC) in $CDCl_3$ at δ = -142.20 ppm. The following abbreviations are used to describe NMR resonances: s (singlet), d (doublet), t (triplet), m (multiplet), br (broad), and app (apparent). Coupling constants (*J*) are reported in Hz. Liquid chromatography followed by high-resolution mass spectroscopy (LC-HRMS) analysis from electrospray ionization (ESI) was carried out on a Waters Acquity-Xevo G2-XS QTof instrument. Observed rotation, α_{obs}, was measured in a standard glass cell (100 mm, 1 mL) using a sodium D-line lamp at 20 ºC in a PerkinElmer Model 241 Polarimeter. Specific rotation [α] was calculated based on $[\alpha]^{20}_{D}$ = $(\alpha_{obs})/[(g_{sample}$ in 1 mL) x 1 dm].

ThioPA (DiC 18:1) was prepared on the basis of Bonnel *et al.* (1) with modifications. To a dry 5-mL round-bottom flask, thiophosphoryl chloride (53 μL, 0.530 mmol, 6.5 equiv) in 1.0 mL of DCM was added under a dry nitrogen atmosphere. The flask was then cooled to -30 °C externally with a mixture of dry ice and acetonitrile. Pyridine (43 μL, 0.530 mmol, 6.5 equiv) in 1.0 mL of DCM was added dropwise to the solution while stirring vigorously. After 15 minutes of mixing, 1-2-dioleoyl-*sn*-glycerol (50 mg, 0.081 mmol, 1.0 equiv) in 1.5 mL of DCM was added dropwise. The reaction mixture was then allowed to warm to room temperature (RT) and stirred for 24 hours. The resulting mixture was determined to primarily contain the thiophosphatidyldichloride product based on TLC analysis (molybdenum blue stain) and concentrated under reduced pressure and re-dissolved in 3.0 mL of THF. The solution was then cooled to 0 °C, treated with 0.50 mL of pyridine/water (1:1 v/v) dropwise, and stirred vigorously for 28 hours. The resulting mixture was diluted with chloroform (5 mL) and dried over sodium sulfate. This crude mixture was then filtered and the filtrate was concentrated to an oil under reduced pressure. The product was purified by column chromatography using an eluent system of $CHCl_3$/MeOH/7 M NH_4OH with a step gradient from 100:0:0 to 65:30:5 v/v/v. Product fractions were collected and concentrated, affording 44 mg (74% isolated yield) of **ThioPA** as a clear film.

^{1}H NMR (500 MHz, $CDCl_3$): δ 5.37–5.30 (m, 4H), 5.27–5.23 (m, 1H), 4.40–4.38 (d, *J* = 10.5 Hz, 1H), 4.20, (m, 1H), 4.04–3.99 (br s, 1H), 3.96–3.92 (br s, 1H), 2.36–2.29 (m, overlapped, 4H), 2.01 (m, 8H), 1.59 (br s, 4H), 1.29–1.27 (m, 40H), 0.88 (app t, *J* = 6.7 Hz, 6H).

^{13}C NMR (126 MHz, $CDCl_3$): δ 173.2, 172.9, 129.2 (overlapped), 128.87, 128.85, 69.9, 69.8, 62.8, 62.3, 33.6, 33.4, 31.1 (overlapped), 29.05, 29.03, 29.0 (overlapped), 28.7 (overlapped), 28.6

(overlapped), 28.56 (overlapped), 28.54 (overlapped), 28.53, 28.51, 28.5, 28.4, 28.2, 26.4, 24.2, 24.1, 21.9 (overlapped), 13.1 (overlapped).

31P NMR (201 MHz, $CDCl_3$): δ -89.41.

HRMS (ESI) m/z: Calculated for $C_{39}H_{72}O_7PS^-$, $[M – H]^-$, requires 715.4742; found 715.4758.

[α]$^{20}_{D}$ = +11.8º (c = 2.0 g/100mL, 0.020 g/mL, $CHCl_3$).

REFERENCE

1. Bonnel S. I., Y.-P. Lin, M. J. Kelley, G. M. Carman, J. Eichberg. 1989. Interactions of thiophosphatidic acid with enzymes which metabolize phosphatidic acid. Inhibition of phosphatidic acid phosphatase and utilization by CDP-diacylglycerol synthase. *Biochim. Biophys. Acta* **1005:** 289-295

^{1}H NMR Spectrum

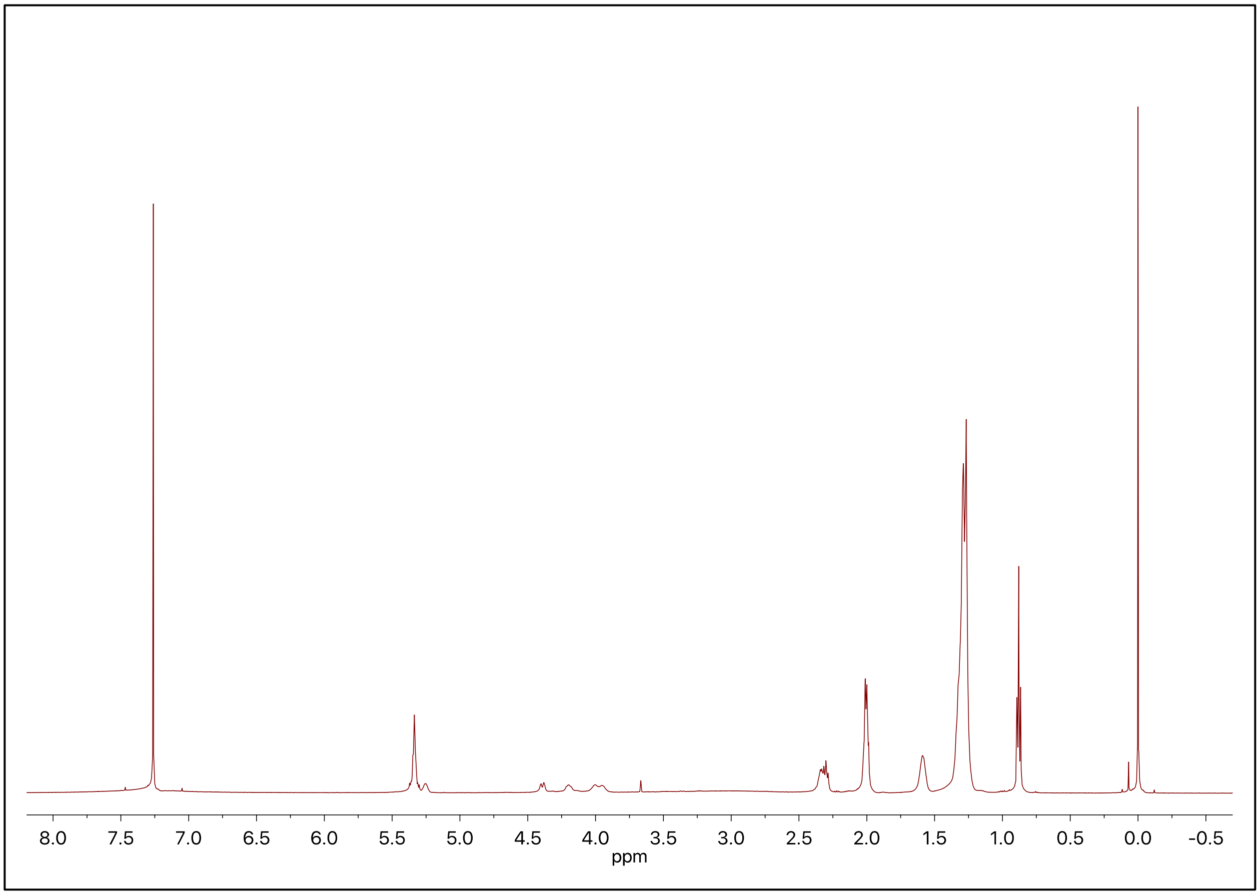

^{13}C NMR Spectrum

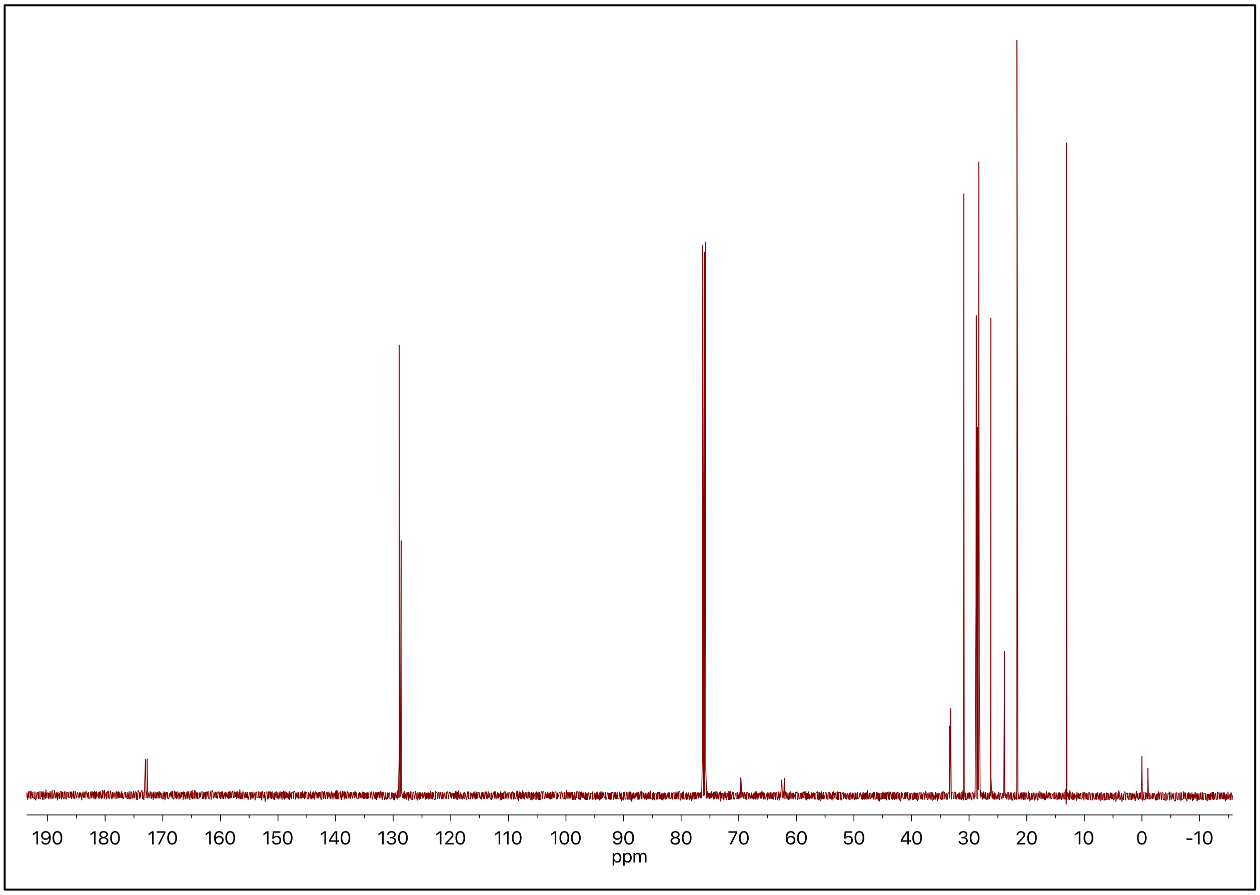

^{31}P NMR Spectrum

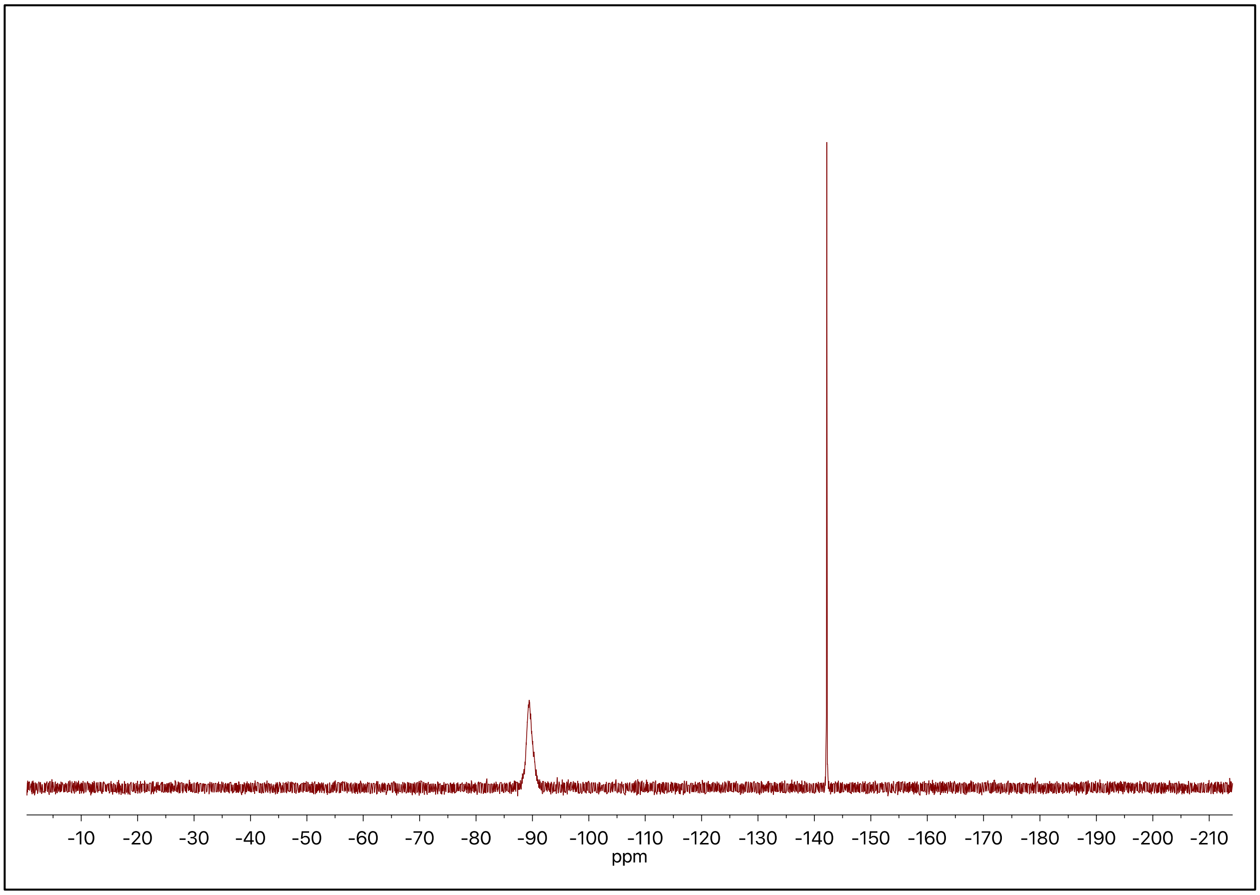

Supplement: Supporting Information [file mmc1.pdf]
